# Rituximab in Connective Tissue Disease-Associated Interstitial Lung Disease: From Immunopathogenesis to Therapeutic Implications

**DOI:** 10.3390/ijms27010046

**Published:** 2025-12-20

**Authors:** Dimitrios Chatzis, Aggelos Banos, Antonis Fanouriakis, Theodoros Karampitsakos, Vasilios Tzilas

**Affiliations:** 1 2nd Pulmonary Medicine Department, University Hospital “Attikon”, Athens Medical School, National and Kapodistrian University of Athens, 104 31 Athens, Greece; chatzisdimit@gmail.com; 2 Rheumatology Unit, University Hospital “Attikon”, Athens Medical School, National and Kapodistrian University of Athens, 104 31 Athens, Greece; abanos@med.uoa.gr (A.B.); afanour@med.uoa.gr (A.F.); 3 Laboratory of Autoimmunity and Inflammation, Center for Clinical, Biomedical Research Foundation, Experimental Surgery and Translational Research, Academy of Athens, 104 31 Athens, Greece; 4 First Department of Pulmonary, Critical Care and Sleep Medicine, Hospital for Thoracic Diseases “SOTIRIA”, National and Kapodistrian University of Athens, 104 31 Athens, Greece; thodoriskarampitsakos@gmail.com

**Keywords:** Rituximab, connective tissue diseases, interstitial lung disease, rheumatoid arthritis, systemic sclerosis, idiopathic inflammatory myopathies, B cell, CD20, autoimmune disease, monoclonal antibody

## Abstract

Connective tissue disease-associated interstitial lung disease (CTD-ILD) comprises a heterogeneous group of immune-mediated pulmonary disorders with significant morbidity and mortality. The pathogenesis involves complex interactions of autoimmunity, chronic inflammation, and fibrosis. B cells play a central role in these processes through antigen presentation, autoantibody production, cytokine secretion, and the formation of ectopic lymphoid tissue within the lung parenchyma. Rituximab (RTX)—a chimeric anti-CD20 monoclonal antibody—depletes B cells and has emerged as a promising therapeutic agent for CTD-ILD. This review comprehensively presents the immunopathogenic mechanisms underlying CTD-ILD, elaborating on the multifaceted mode of action of RTX and summarizing the evolving clinical evidence.

## 1. Introduction

Connective tissue diseases (CTDs), including systemic sclerosis (SSc), rheumatoid arthritis (RA), and idiopathic inflammatory myopathies (IIMs), frequently manifest lung involvement, leading to interstitial lung disease (ILD). ILD represents a major cause of morbidity and mortality in these patients. The pathogenesis of CTD-ILD is multifactorial, involving complex interactions between the immune and stromal compartments. B-cells play a central role by promoting autoantibody production, antigen presentation, cytokine secretion, and the formation of ectopic lymphoid structures (ELS) within the lung parenchyma, a phenomenon well-described in several CTDs, such as Sjögren’s disease and RA. Rituximab (RTX) is a chimeric murine–human monoclonal antibody that specifically binds to the CD20 surface antigen expressed on pre-B and mature B-cells, sparing pro-B-cells and plasma cells. It mediates B-cell depletion through several overlapping and complementary mechanisms, including antibody-dependent cellular cytotoxicity (ADCC), complement-dependent cytotoxicity (CDC), and the direct induction of apoptosis. Beyond its depleting effect, RTX exerts broader immunomodulatory actions: it suppresses antigen presentation and T-cell co-stimulation, rebalances the cytokine milieu, disrupts ectopic lymphoid structures in inflamed tissues, and indirectly modulates fibroblast activation. The scope of this review is to describe the mechanisms of action of RTX and summarize its clinical application across CTD-ILD, integrating current mechanistic insights with therapeutic outcomes.

## 2. The Central Role of B Cells in the Pathogenesis of CTD-ILD

B cells play multifaceted roles in the pathogenesis of CTD-ILD through the production of autoantibodies, antigen presentation, and T-cell presentation, recruitment of pro-inflammatory cells like macrophages, and secretion of cytokines.

### 2.1. Role of Immunoglobulin Gene Rearrangements in B-Cell Development

A central mechanism by which B cells contribute to the pathogenesis of CTD-ILD is through the production of autoantibodies. In this regard, a critical step in B-cell development is the generation of the B-cell receptor (BCR). The rearrangement of the Ig heavy- and light-chain genes is indispensable for B-cell development and maturation. The immunoglobulin heavy chain consists of roughly 40 functional variable (V) segments, 9 diversity (D) segments, 6 joining (J) segments, and a constant region for each subclass of immunoglobulin heavy chains (mu, gamma, alpha, delta, and epsilon). Light-chain genes are organized similarly but lack D segments [1]. A complete immunoglobulin molecule is formed by two heavy and two light chains, allowing for the assembly of the mature B-cell receptor and signaling towards cessation of light-chain gene rearrangement. Also, the multiple combinations of segments and chains that are used for a BCR to be produced ensure a vast diversity of BCRs, capable of recognizing different antigens [2].

### 2.2. B-Cell Development and Maturation

B-cell development commences with the migration of multipotent progenitor cells (MPPs) first into the fetal liver and then into the bone marrow. MPPs differentiate into the common lymphoid precursors (CLPs), which produce the common lymphoid 2 progenitor of the B-cell lineage (pro-B Cell) [1]. The pro-B Cell is instructed by stromal bone marrow cells to induce the development of B cells, orchestrated by interleukin (IL)-7, Fms-like tyrosine kinase 3 ligand (Flt3-L), and PAX5 (paired box gene 5) [3]. Once immunoglobulin (Ig) heavy-chain V-D-J recombination is completed, a pre-B Cell is formed. Simultaneously, a number of markers appear for the first time, including CD20, which functions as a Ca++ conductive ion channel, regulating B-cell activation and proliferation [4]. The transition from the pre-B-cell stage to the immature B-cell stage is demarcated by the successful expression of a complete IgM antibody on the cell surface. Immature B cells egress from the bone marrow and enter circulation as transitional B cells, populating the blood and secondary lymphoid organs. These transitional cells are direct precursors of more terminally mature pre-immune B-cell subtypes, namely follicular (FO), marginal zone (MZ), germinal center (GC), and memory B cells [5]. Importantly, CD20 is expressed on ~95% of B cells in peripheral blood and secondary lymphoid organs [6]. Other important B-cell-specific markers are (1) CD19, which is expressed widely in the B-cell lineage and participates in intracellular signal transduction, and (2) CD21, a C3d and Epstein–Barr virus receptor, interacting with CD19 to transduce inflammatory signals [7]. Mature B cells are subcategorized into CD5- (or “conventional” or B-2 cells) and CD5+ (B-1a and B-1b cells) [8].

### 2.3. Antigen Presentation, T-Cell Activation, and Auto-Antibody Production

B-cell activation is initiated when surface receptors of B cells bind to specific antigens. Then, naïve B cells (antigen-agnostic) become activated and differentiate into antibody-producing plasma cells and memory B cells. A portion of plasma cells migrates to the bone marrow, where they persist as long-living cells, producing antibodies even in the absence of antigens. There are two modes of B-cell activation depending on the nature of the antigen. Non-protein antigens, such as lipids and nucleic acids, stimulate antibody production in the absence of T cells and are referred to as thymus-independent (TI) antigens. In contrast, the antibody response to protein antigens requires both B and T cells, characterized as thymus-dependent (TD) antigens.

### 2.4. T-Cell-Dependent B-Cell Activation

T-cell-dependent B-cell activation occurs in two anatomically different phases. The early phase takes place in lymphoid organs and involves B-cell proliferation, initial antibody secretion, and isotype switching. In the late phase, affinity maturation and B-cell memory formation occur in GCs, within lymphoid follicles. Within 1–2 days of antigen exposure, naïve CD4+ T cells become activated through antigen presentation by professional antigen-presenting cells in the T-cell area, while B cells recognize the same antigen and move to the T-cell area. Antigen-activated T and B cells interact at the follicles and T-cell area 3–7 days post-antigen exposure.

T-cell-mediated activation of B-cells is established when antigen-specific B cells bind antigens via Ig receptors, enhancing the expression of co-stimulatory molecules on their surface. Concomitantly, B cells internalize the antigen-bound receptor through endocytosis, and the fragments are presented via MHC Class II to cognate CD4+ T cells. B cells express B7-1 and B7-2 during antigen processing, which bind via CD28 on T cells, leading to proliferation. T cells also express CD40L, a partner of CD40 on the B-cell surface, resulting in the transcription of immunoglobulin genes and release of cytokines [9].

In the late phase of T-cell-dependent B-cell activation, GCs form within 4–7 days of antigen exposure at the borders of B-cell follicles and T-cell zones. GCs are transient structures where affinity maturation and memory B cell and long-lived plasma cell generation occur. Each GC arises from antigen-specific B-cell clones [10]. Within GCs, follicular dendritic cells present antigens and express complement receptors (CD35/CR1, CD21/CR2, CD11b/CD18), Fc receptors, and CD40L, driving B-cell proliferation in the dark zone. Affinity maturation follows, driven by the somatic hypermutation of Ig genes and the selection of high-affinity clones. Following maturation, some B cells become memory cells for rapid secondary responses, while others become long-lived plasma cells.

### 2.5. B Cells and Cytokine/Chemokine Secretion

Human B Cells secrete various cytokines, including pro-inflammatory (e.g., IL-6), immunosuppressive (e.g., IL-10), and chemokines (e.g., CCL22) to regulate immune responses.

The major chemokines produced by naïve B cells upon activation are CCL22 and CCL17. These two attach to the same receptor (CCR4), expressed in Th2 cells, promoting their recruitment by activated B cells and cytokine secretion, such as IL-4 [11]. CCL3 and CCL4 attract T lymphocytes and monocytes, enhancing their infiltration into various tissues [12].

B-cell cytokine production is highly in tandem with their activation status. After activation via BCR cross-linking and CD40 stimulation, B cells produce effector cytokines, like IL-6 and TNF-α. If B cells receive only CD40 stimulation, they conversely produce immunoregulatory cytokines, such as IL-10, and dampen the expression of effector cytokines [13]. Thus, B cells can be broadly subdivided into two subsets: cytokine-producing B cells that propagate (effector cells) or regulate (regulatory cells) immune responses. Effector cells differentiate into two distinct cytokine profiles, known as B effector (Be)1 and (Be)2, secreting IFNγ, TNF-α, and IL-12 or IL-4 and IL-10, respectively [14]. On the other hand, B-regulatory cells (B-regs) suppress the immune response mainly via IL-10, but also TGF-β and IL-35 [15,16,17].

### 2.6. Formation of Ectopic Lymphoid Structures

B cells play a central role in forming Ectopic Lymphoid Structures (ELSs), also called tertiary lymphoid structures (TLSs), which develop in non-lymphoid tissues in response to chronic inflammation, infection, or autoimmunity. On the contrary, TLSs lack capsular and afferent lymphatic vessels, and they disappear after antigen clearance. ELSs are characterized by B-cell and T-cell segregation, partly via ectopic expression of lymphoid-associated chemokines CXCL13, CCL19, and CCL21. They are also defined by high endothelial venules (HEVs) and often by the presence of CD21+-follicular-dendritic-cell (FDC) network, which sustains the local humoral B-cell response [18].

As mentioned before, inflammatory triggers such as those seen in autoimmune diseases may cause the formation of ELSs in targeted tissues. A typical paradigm is the synovial tissue in RA [19]. The chemoattractant signaling pathway involves several homeostatic lymphoid chemokines, such as CXCL13, CCL19, CCL21, and CXCL12 [20]. At the same time, inflammatory cytokines are also crucial for the lymphoid emergence in autoimmune diseases, including IL-17, IL-21, IL-22, IL-23, IL-27, and TNF-α [21,22]. ELSs in autoimmune conditions act like GCs, favoring the proliferation and maturation of B cells via an antigen-driven selection process. Their differentiation into plasma cells, achieved by class switch recombination, results in high-affinity antibodies [23,24].

## 3. Mechanism of Action of Rituximab

RTX is an immunoglobulin G1 (IgG1) monoclonal antibody (mAb) that targets CD20, a protein expressed on the surface of most B cells. This chimeric antibody, composed of both mouse variable regions, carries the amino acid sequences specific for CD20 and human constant regions, which are responsible for its functional properties. RTX acts primarily by depleting CD20-positive B cells. CD20 is a phosphoprotein expressed on the surface of almost all B cells, functioning as a store-operated calcium channel following the ligation of the B-cell receptor for antigens. Moreover, its association with the Src family tyrosine kinases suggests the involvement of CD20 in transmembrane signaling [4,25]. CD20 is an exclusive B-cell marker, emerging at the pre-B-cell stage while absent at earlier or later stages of B-cell differentiation. Importantly, plasma cells lack CD20 expression. This antibody mediates B-cell depletion through several overlapping and complementary mechanisms (summarized in Table 1) [26,27]:

- Antibody-Dependent Cellular Cytotoxicity (ADCC);
- Complement-Dependent Cytotoxicity (CDC);
- Direct induction of apoptosis.

### 3.1. Antibody-Dependent Cellular Cytotoxicity

The therapeutic efficacy of RTX is largely attributed to its ability to recruit immune effector cells through antibody-dependent cellular cytotoxicity (ADCC). The human part of RTX (Fc portion) binds to Fcγ receptors, particularly FcγRIIIa (CD16), expressed on NK cells and initiates a cascade of cytotoxic events leading to B-cell depletion [28,29]. NK-cell engagement results in the release of perforin and granzymes, inducing apoptosis in RTX-opsonized B cells, a mechanism strongly supported by animal models in which Fcγ receptor blockade abrogates therapeutic benefit [30]. The interaction between RTX and FCγRIIIa is of crucial importance. Some authors have shown that the responses to RTX are related to FCγRIIIa-receptor polymorphisms. In particular, FcRIIIa homozygous for valine at position 158 of the protein (VV) has a higher affinity for IgG1 than does FcRIIIa with phenylalanine at that position (VF or FF) [31,32]. Beyond NK cells, macrophages and neutrophils contribute to ADCC through FcγR-mediated cytotoxicity and phagocytosis [33]. Finally, RTX-opsonized B cells act as decoy entities that effectively divert monocytes or macrophages from binding to tissue-associated immune complexes, implying an alternative mechanism of RTX action in the autoimmune context [34]. In addition, the effects of RTX also extend to innate immune cells. Ex vivo studies demonstrated that activated macrophages effectively eliminated chronic lymphocytic leukemia cells in the presence of RTX, a process mediated through antibody-dependent cellular cytotoxicity [35].

### 3.2. Complement-Dependent Cytotoxicity

Complement-Dependent Cytotoxicity (CDC) represents another major pathway underlying RTX-mediated B-cell depletion, as observed in data from trials associated with malignant B cells. RTX binds to C1q, fueling the activation of the classical branch of the complement cascade [36]. In addition, synergism between CDC and ADCC is founded on the ability of the complement system to promote inflammation and propagate the activation of innate effectors. During complement activation, C3a and C5a function as anaphylatoxins to recruit effector cells and activate them through playing the rheostat of FcγR expression. Additionally, CR3 binding to iC3b ligands amplifies ADCC [33,37,38]. Some frequently observed adverse effects during RTX therapy are infusion reactions (fever and rashes), which are associated with enhanced complement activation products (C3b/c and C4b/c) linked to triggering of the complement cascade [39].

### 3.3. Rituximab and Direct Induction of Apoptosis

In addition to ADCC and CDC, RTX mediates B-cell depletion through the direct induction of apoptosis, a mechanism that reflects the intrinsic signaling consequences of CD20 ligation. Main pro-apoptotic pathways triggered by RTX include caspase-dependent cascades [40,41]. Three different pathways are implicated: (a) activation of Src family tyrosine kinases (Lyn, Fyn, and Lck), (b) activation of Fas-apoptotic signaling, and (c) inhibition of major survival pathways, such as p38 MAPK, ERK1/2, NF-κB, and Akt, studied mainly in hematological malignancies. Deans et al. showed that CD20 is associated with lipid rafts, enriched in Src family tyrosine kinases and other signaling effectors [42]. RTX binds to CD20, rearranging lipid rafts, transactivating Src tyrosine kinases, and signaling downstream towards apoptosis.

### 3.4. Immunomodulatory Effects

Beyond B-cell depletion, the immunomodulatory effects of RTX expand to (a) decreased autoantibody production, (b) reduced antigen presentation, and (c) disruption of ectopic lymphoid structures.

### 3.5. Decreased Autoantibody Production

The autoantibody decline following RTX treatment varies depending on the disease and the antibody type. In RA, early studies demonstrated that B-cell depletion with RTX led to significant clinical improvement, followed by decreased levels of rheumatoid factor (RF) [43]. Another study assessed the levels of C-reactive protein (CRP), antimicrobial antibodies, autoantibodies (such as IgA-, IgM-, and IgG-class rheumatoid factors (RF)), and antibodies to cyclic citrullinated peptide (anti-CCP) in 22 patients with RA treated with RTX. Following B-lymphocyte depletion, a favorable clinical response was observed, accompanied by a significant reduction in CRP and autoantibodies. The time to relapse after B-lymphocyte return was often prolonged and variable (range 0–17 months). However, relapse was closely paired with the peak of at least one autoantibody [44]. Similarly, a randomized, double-blind, controlled study in patients with active RA showed that treatment with RTX is associated with the serum levels of autoantibodies being proportionally more affected than total immunoglobulin levels. This suggests that these autoantibodies represent partly a product of short-lived plasma cells (that are not renewed following the depletion of the B-cell precursors) [45].

Additionally, in antineutrophil cytoplasmic antibody (ANCA)-associated vasculitis (AAV) patients treated with RTX, B lymphocytes were zeroed, and ANCA titers were dampened substantially [46]. Subsequent prospective trials confirmed that reductions in ANCA titers often paralleled clinical improvement. Notably, disease relapse was associated with B-cell reconstitution and renewed ANCA positivity, underscoring the dynamic link between B-cell recovery and pathogenic autoantibody production [47].

In SLE, the landscape is more heterogeneous. A study reported the preservation of immunoglobulin levels and protective antibodies, but a reduction in anti-dsDNA antibody titers, often independently of the clinical response [48]. Likewise, a cohort of 16 patients with refractory SLE was assayed for anti-nucleosome, anti-double-stranded DNA (anti-dsDNA), anti-extractable nuclear antigen, anti-tetanus toxoid, and pneumococcal capsular polysaccharide-related antibodies for at least 1 year following anti-CD20 therapy. Anti-nucleosome and anti-dsDNA antibodies decreased, but with considerable interpatient variability, while levels of other autoantibodies and antimicrobial antibodies remained mainly unchanged. A trend towards a sustained low titer of anti-dsDNA antibodies was apparent in patients with clinical amelioration for >1 year. Evidently, B-cell clones committed to anti-nucleosome and anti-dsDNA antibodies have a relatively rapid turnover compared with clones producing other antibodies [49].

### 3.6. Reduced Antigen Presentation

B lymphocytes function as professional antigen-presenting cells (APCs), acting at the crossroads of innate and adaptive immunity. In addition to their well-established role in antibody production, B cells provide co-stimulatory signals and cytokines essential for T-cell activation and polarization. RTX induces B-cell depletion and thereby profoundly alters antigen presentation pathways. The mechanistic insights derived from its clinical use in autoimmune and lymphoproliferative disorders highlight not only direct effects on B cells but also downstream consequences for T-cell co-stimulation, regulatory T-cell expansion, and innate immune function.

B cells display abnormal signaling, expressing aberrant cell-surface markers (e.g., CD40 ligand), thereby activating T cells through cognate interactions and helping organize and regulate inflammatory immune responses. This suggests an additional path for B cells, independent of autoantibody production. In this respect, it has been demonstrated that RTX treatment not only reduces B-cell number and autoantibody levels but also downregulates CD40 and CD80 on B cells, affecting a possible disturbance of T-cell activation through these co-stimulatory molecules [50]. Similarly, remission of proliferative lupus nephritis after B-cell depletion was preceded by the reduced expression of CD40 ligand on T cells, indicating a disruption of the critical CD40–CD40L axis that facilitates T-/B-cell crosstalk. Moreover, the expression of T-cell activation markers CD69 and HLA-DR was significantly lowered in partial remission and further decreased in complete remission [51]. Lastly, a study demonstrated that a single dose of RTX does not deplete B cells in secondary lymphoid organs but modulates their function. After stimulation of allogeneic T cells with LN-derived B cells from RTX-treated patients, proliferating T cells exhibited lower production of IL-17, reflecting reduced expression of antigen-presenting and costimulatory molecules by the residual B cells [52].

### 3.7. Rituximab and Modulation of Cytokine Milieu

RTX exerts profound effects on the cytokine environment, reshaping the balance between pro-inflammatory and regulatory mediators that govern immune homeostasis. By depleting CD20^+^ B cells and modifying their interactions with T cells and macrophages, RTX indirectly influences cytokine production and signaling.

One of the most consistent findings is that RTX treatment reduces the levels of pro-inflammatory cytokines, such as IL-6, IL-7, and TNF-α, while restoring anti-inflammatory mediators like IL-10. In a study of 45 patients treated by RTX, IL-2, IL-6, IL-7, and IL-10 decreased after treatment. The reduction in IL-2, IL-6, and IL-7 is consistent with the broader observation that B-cell depletion disrupts the chronic inflammatory circuits [53]. Furthermore, another study showed that RTX reduced serum IL-15, along with IL-17, in RA patients, which is associated with clinical improvement [54]. Serum matrix metalloproteinase (MMP) and tissue inhibitor of metalloproteinases-1 (TIMP-1) levels in patients with active RA not responding to anti-tumor necrosis factor (anti-TNF) therapy appear substantially decreased after RTX therapy [55]. The impact of RTX on T-cell-derived cytokines in RA is also substantial. T-cell-driven inflammation in the synovial membrane in RA occurs via cytokines provided by B cells, such as IFN-gamma and IL-1β, two cytokines inhibited by anti-CD20 therapy [56].

Another dimension of cytokine modulation is observed in other autoimmune syndromes. A study related to primary Sjögren’s disease demonstrated that a subset of IL-17-secreting T cells expressing CD20 is directly targeted by RTX, resulting in reduced IL-17 production and improved glandular function [57]. In SLE, the treatment led to a decrease in TNF-α levels along with normalization of the Th1/Th2 cytokine ratio [58]. Additional evidence of the capacity of RTX to remodel the inflammatory milieu arises from a study about atopic eczema, exhibiting a reduction in IL-5 and IL-13 [59].

Innate immune modulation also contributes to cytokine rebalancing. A study assessed changes in macrophage phenotype and function after RTX-induced B-cell depletion in patients with RA. Investigators observed altered macrophage cytokine secretion patterns characterized by reduced TNF-α and IL-1β production and enhanced expression of anti-inflammatory mediators, such as IL-10 [60]. Moreover, cytokine release can also mediate some of the infusion-related reactions seen with RTX. A study of 37 patients with chronic lymphocytic leukemia treated with RTX identified elevated interferon-inducible protein-10 (IP-10) levels during early infusions, correlating with transient flu-like symptoms [61].

### 3.8. Rituximab and Ectopic Lymphoid Structures

The role of RTX in modulating ELS has been increasingly recognized in chronic autoimmune and inflammatory disorders. A compelling clinical example is provided by a case report, which described two cases of patients with Common Variable Immune Deficiency (CVID)-associated ILD, with biopsies demonstrating loosely organized ELS. Following RTX monotherapy, radiological and histological improvement was observed, with the partial regression of peribronchial lymphoid infiltrates and granulomatous inflammation. This case highlights how RTX can target the lymphoid component of chronic interstitial inflammation, suggesting that ELS may represent therapeutic targets for B-cell-directed therapy [62]. The broader relevance of this mechanism is reflected in autoimmune conditions where ELS formation is central to pathogenesis. In a study that assessed the effect of the drug on T- and B-cell subsets, lymph node biopsies were obtained from 14 patients with RA before and 4 weeks after the first infusion. Despite a significant fall in naïve and unswitched memory B cells, switched memory B cells persisted. In the T-cell compartment, early-activated tissue-resident T cells were decreased after RTX treatment, while late-activated T cells remained stable. Remodeling of lymphoid microarchitecture and disruption of the cellular interactions are necessary for the maintenance of ELS [63]. Additionally, ELS in the rheumatoid synovial joints seem to sustain autoreactivity against locally expressed autoantigens. In particular, ELSs produce autoantibodies against specific antigens such as HSP60, a protein located on the surface of fibroblast-like synoviocytes. An apparent reduction in synovial Heat Shock Protein 60 (HSP60) gene expression followed B-cell depletion with RTX, correlated with treatment response and collapse of ELS [64]. In salivary glands in patients with Sjögren’s disease, ELSs play a key role in local autoantibody generation and lymphoepithelial lesions. A recent study showed that levels of NKp30 (an NK cell-specific activating receptor regulating the cross-talk between NK and dendritic cells) are significantly increased in salivary glands with organized ELS. In this study, RTX markedly reduced NKp30 expression and disrupted the organization of B-/T-cell zones within ELS [65]. Supportive data come from a prospective, multicenter, follow-up study of 41 patients with primary Sjögren’s disease treated with RTX. The authors observed that RTX reduced glandular infiltrate and altered B/T compartmentalization, and ultimately, the formation of ELSs and germinal center-like structures in labial minor salivary gland (MSG) biopsies from patients with primary Sjögren’s disease [66].

### 3.9. Rituximab and Effect on Fibroblast Activation

Beyond its established immunomodulatory role, RTX exerts secondary effects on tissue remodeling by influencing fibroblast activation and fibrogenesis. A multicenter study of patients with SSc evaluated fibroblast activation by performing skin biopsies at baseline and at 3 and 6 months post-treatment. Investigators demonstrated that skin fibroblasts after a single RTX cycle showed significantly reduced type I collagen mRNA levels and the normalization of fibroblast phenotype. This effect coincided with a decrease in anti-Platelet-Derived Growth Factor Receptors (PDGFR) autoantibodies, which are known to stimulate fibroblast proliferation and collagen synthesis [67]. Similarly, an open-label clinical and histopathological study reported histological regression of dermal fibrosis after therapy with RTX. Post-treatment biopsies revealed decreased dermal thickness and myofibroblast density, further supporting a direct anti-fibrotic effect [68]. Moreover, a long-term follow-up study of patients with diffuse SSc, treated with RTX, confirmed sustained improvements in both lung function and dermal thickness with prolonged B-cell depletion. A reduction in histological myofibroblast score was also seen [69]. Furthermore, a study tested if RTX mediates its antifibrotic effects in patients with SSc via Dickkopf-1 (Dkk-1), an inhibitor of the Wnt pathway. The Wnt signaling pathway is critically involved in orchestrating cellular functions such as proliferation, migration, survival, and cell fate determination during development [70]. These observations are further supported by recent data in patients with RA and psoriatic arthritis (PsA), demonstrating that serum levels of DKK1, Wnt5a, and β-catenin were significantly elevated compared with healthy controls, and closely correlated with disease-activity indices and bone-remodeling biomarkers [71]. Dkk-1 expression was immunohistochemically assessed in skin biopsies obtained from 11 patients with SSc. RTX correlated with the upregulation of Dkk-1 skin expression. In contrast, TGFβ was significantly attenuated following treatment, specifically in patients with Dkk-1 upregulation [72].

## 4. Clinical Evidence for Rituximab in CTD-ILD

### 4.1. Connective Tissue Disease-Associated Interstitial Lung Diseases as a Group

RTX has emerged as a promising therapy for CTD-ILD, especially in cases refractory to conventional immunosuppressive treatment, based on the results of two pivotal studies. The RECITAL study [73] was a double-blind, double-dummy, phase 2b RCT assessing the superiority of RTX compared to cyclophosphamide (CYC). It included 101 patients from 18 to 80 years of age (38% patients with scleroderma, 46% patients with idiopathic inflammatory myopathies, and 16% patients with mixed connective tissue disease). Participants were randomly allocated in a 1:1 ratio to receive either RTX (administered intravenously at 1000 mg on weeks 0 and 2) or CYC (given intravenously at 600 mg/m^2^ of body surface area every four weeks for six total infusions). The main outcome measure was the change in FVC from baseline to week 24. Both treatments demonstrated comparable benefits, leading to improvements in FVC at weeks 24 and 48, in six-minute walk distance at 24 weeks, and in quality of life at 24 and 48 weeks. However, the use of RTX was associated with fewer adverse events and lower glucocorticoid exposure. Notably, the strongest treatment responses to both CYC and RTX were observed in the subgroup of patients with IIM-ILD. Consequently, evidence from studies, such as RECITAL, suggests that treatment effects are not uniform across subtypes. CTD-ILDs represent a heterogeneous group of disorders with distinct pathogenetic mechanisms and variable responsiveness to therapy. Therefore, therapeutic outcomes should not be over-generalized, and the efficacy of RTX must be interpreted within the clinical and biological context of each underlying CTD.

The EVER-ILD study [74] was a randomized, double-blind, two-parallel group, placebo-controlled trial, including 122 patients with CTD-ILD or idiopathic interstitial pneumonia (with or without autoimmune features) and a non-specific interstitial pneumonia (NSIP) pattern, that were randomly assigned in a 1:1 ratio to receive RTX (1000 mg) or placebo on day 1 and day 15 in addition to MMF (2 g daily) for 6 months. The primary endpoint was the percentage change in predicted FVC from baseline to 6 months. The combination of RTX and MMF was superior to MMF alone. Importantly, this effect was lost at 12 months, supporting the continuation of treatment with RTX [75].

Finally, in a retrospective study, 49 patients with CTD-ILD [76] treated with RTX resulted in an improved FVC and stabilized DLco at 1 year. The positive effect was stronger in patients with an imaging pattern of NSIP compared to UIP, although the latter group also exhibited an increase in functional parameters.

The clinical studies of RTX for the treatment of CTD-ILD are summarized in Table 2.

### 4.2. Rheumatoid Arthritis

A growing body of evidence supports the efficacy of RTX in RA-ILD, demonstrating its ability not only to stabilize but also, in some cases, improve lung function. Md Yusof et al. [77] conducted a 10-year single-center observational study to evaluate the effect of RTX in patients with RA-ILD. Progression was defined by a decrease in FVC > 10% predicted, diffusing capacity for carbon monoxide (DLCO) > 15% predicted, radiological deterioration in HRCT, or death from progressive ILD. Of the 44 patients in this cohort, 23 (56%) were stable and 7 (16%) improved, while 14 (32%) showed progression, with 9 deaths (16%) due to progressive ILD. UIP (Usual Interstitial Pneumonia) pattern, previous progression, and DLco-predicted lung progression post-RTX were observed. Similarly, a longitudinal multicenter study of 68 patients in Spain [78], using data from the NEREA registry, showed that patients exposed to RTX had a higher probability of remaining free of functional impairment compared to other therapies. It should be noted that in this study, functional impairment was defined as a relative decline of FVC (Forced Vital Capacity) ≥ 5%. The RTX non-exposed group was treated with azathioprine (AZA) and mycophenolic acid (MMF) in descending order of frequency. In a retrospective multicenter study by Kelly et al. [80], patients with RA-ILD receiving RTX as their first biologic factor had longer three- (92%), five- (82%), and seven-year (80%) survival than those whose first biologic treatment was a TNF inhibitor (82%, 76%, and 64%, respectively). This evidence was supported by Druce et al. [81] in a prospective observational comparative study, which found that patients with RA-ILD who received RTX had lower mortality rates than those who received TNF inhibitors. RTX has also been found to be comparable to CYC in increasing FVC and improving quality of life parameters in RA-ILD patients. Saleem et al. [82] performed a randomized open-label study in 95 RA-ILD patients, assigning them randomly to RTX and CYC. Both groups demonstrated an improvement in FVC from baseline, with no statistically significant difference at 24 and 48 weeks.

An observational study from Narváez et al. [83] assessed the effectiveness of RTX as a rescue therapy in 31 patients with progressive RA-ILD despite treatment with glucocorticoids and conventional synthetic Disease Modifying Anti-Rheumatic Drugs (DMARDs) or other immunosuppressants. After one year of treatment, RTX reversed the decline in both FVC and DLco. Among patients who completed two years of RTX therapy, the beneficial effect observed during the first year was sustained over the second year of treatment. It is important to emphasize that the beneficial effect of RTX was observed in both the UIP and non-UIP groups. This result is in concordance with the findings of a retrospective study in 212 patients with RA-ILD, of whom 92 (43.4%) were treated with AZA, 77 (36.3%) with MMF, and 43 (20.3%) with RTX. All three treatments led to the improvement of FVC and DLco at 1 year when compared with the pre-treatment pulmonary function trend before additional immunosuppression. Again, a beneficial effect was observed regardless of the underlying imaging pattern, i.e., UIP vs. non-UIP [97].

Finally, Bopana et al. [79] conducted a meta-analysis across a total of 314 RTX-treated RA-ILD cases, with a heterogenous spectrum of disease representative of real-world clinical practice. Patients under RTX treatment exhibited a statistically significant improvement in FVC from baseline by 7.5%, while there was a numerical improvement in DLco by 6.4%.

Based on the above, RTX is a promising therapeutic option in the management of RA-ILD, as existing data support its beneficial role in the stabilization and/or improvement of pulmonary function and in lowering mortality. The drug was included in the recent 2023 ACR/CHEST [98] and ERS/EULAR guidelines [99] as a conditional recommendation not only for refractory cases, but also as first-line treatment for RA-ILD.

### 4.3. Systemic Sclerosis

In SSc, RTX has been used mainly in refractory cases after treatment with MMF or tocilizumab. Two RCTs have compared RTX with CYC. Maher et al. [73] conducted a double-blind, double-dummy, phase 2b RCT, named RECITAL, to compare the two therapies in a group of 110 patients of various connective tissue disease-ILDs, including patients with scleroderma (38%). RTX showed similar efficacy to CYC, and results were consistent in all subgroups of connective tissue diseases. However, safe conclusions for scleroderma can be drawn only by studies having statistical power for this subgroup of patients. Basket trials, such as RECITAL, are advisable for CTD-ILDs to simplify the enrolment of a large sample size. Nonetheless, it should be stated that treating CTD-ILDs as a homogeneous group has caveats. CTD-ILDs share common immune perturbations but also exhibit substantial heterogeneity in terms of lung manifestations. Thus, findings from basket studies may not always be generalizable [73]. Aside from RECITAL, Sircar et al. conducted an open-label RCT [86] in 60 patients with early SSc-ILD. There was an improvement in FVC in the RTX group, while it declined in the CYC group. Also, serious adverse events were more common in the CYC group.

Regarding observational studies, a post hoc analysis of 955 patients with SSc-ILD from the EUSTAR database, which compared different treatments for SSc-ILD (including CYC, tocilizumab, and MMF), revealed no statistically significant differences in FVC trajectories [87]. Daoussis et al. [89] compared the efficacy of RTX plus standard therapy (prednisone, CYC, MMF) with standard therapy alone in 14 patients with SSc-ILD. Eight patients in the RTX group demonstrated a significantly better FVC and DLCO at one year compared to the six patients who received standard therapy alone. Actually, in the latter group, there was a decline in both FVC and DLco over the same period. The improvement in FVC in the RTX group was maintained at the two-year follow-up [69]. This study was followed by a multicenter, open-label study of 51 patients comparing RTX to conventional treatment in SSc-ILD [88]. At the two-year follow-up, patients treated with RTX exhibited a significant increase in FVC, whereas the control group failed to show any improvement. A meta-analysis from Goswami et al. [84] assessed the efficacy of RTX on lung function parameters (FVC and DLCO) in SSc-ILD patients. It included 20 studies and 575 patients. RTX improved FVC from baseline by 4.49% and 7.03% at 6 and 12 months, respectively. DLCO also improved by 3.47% and 4.08% at 6 and 12 months, respectively, while the improvement of FVC at 6 months was greater in the RTX group compared to controls. Interestingly, RTX was associated with fewer infectious adverse events compared with other immunosuppressants. Another systematic review and meta-analysis by Macrea et al. supported the benefits of RTX on pulmonary function in SSc-ILD by significantly improving FVC [85].

The 2023 ACR/CHEST guidelines [98] conditionally recommend RTX as a first-line SSc-ILD treatment option. The ERS/EULAR guidelines [99] conditionally recommend RTX for SSc-ILD, especially in patients with a high risk of progression or severe multiorgan involvement.

In the future, precision medicine approaches might lead to different immunosuppression based on the patient’s endotypes [100]. For example, tocilizumab might be an option in early disease or in patients with high cGAS or IL-6, while patients with other endotypes/phenotypes might be more likely to experience benefit from RTX. Biologically enriched, randomized controlled trials could optimize benefit and concomitantly reduce side effects from unnecessary exposure to non-targeted approaches [101].

### 4.4. Idiopathic Inflammatory Myopathies

Idiopathic Inflammatory Myopathies (IIMs) are a distinct subgroup of CTDs prone to ILD development (IIM-ILD), and encompass various phenotypes, such as dermatomyositis (DM), polymyositis (PM), antisynthetase syndrome (ASyS), and overlap syndrome. ILD is highly prevalent among patients with IIM, affecting approximately 65–75% of cases [102], and is particularly frequent in ASyS, where it occurs in 70–95% of patients [103]. The diagnosis can be particularly challenging, especially in amyopathic cases [104,105], as it can mimic other ILDs [106,107]. IIM-ILD represents an archetypal form of CTD-ILD, characterized by a prominent inflammatory component; therefore, immunomodulatory therapy constitutes a central aspect of its management [108].

Bauhammer et al. retrospectively studied the effect of RTX in 16 patients with ILD due to Jo1(+)-ASyS. RTX was administered to patients with severe ILD or refractory disease to other immunosuppressants [90] and resulted in an improvement of both FVC and DLco. Notably, patients with high levels of anti-Ro52 antibodies exhibited a good response to RTX, but no response to other immunosuppressants, including CYC, cyclosporine, AZA, methotrexate, or leflunomide.

A pilot study from Conticini et al. [91] prospectively assessed the efficacy of RTX as a steroid-sparing treatment in 27 patients with ASyS, including 18 patients with ILD. Prednisone was gradually tapered and eventually discontinued within 6 or 12 months. The majority of patients initiated RTX therapy after exhibiting an inadequate response to other immunosuppressive agents, including MMF and methotrexate. Eight patients with ILD received MMF as initial therapy, with five requiring a switch to RTX because of insufficient benefit. All patients achieved clinical remission by the end of follow-up. Furthermore, Allenbach et al. [92] evaluated the implementation of RTX in 10 patients with ASyS-ILD who were refractory to conventional treatments (prednisone and at least two immunosuppressants). In this open-label phase trial, the improvement of ILD was defined as an increase of 10% in FVC and/or an increase of 15% in DLco, while ILD worsening was defined as a decrease of 10% in FVC and/or 15% in DLco. At 12 months, an improvement of ILD was observed in 5 of 10 patients, stabilization in 4, and worsening in 1. Glucocorticoid doses decreased from 52.5 mg/d to 9 mg/d (range, 7–65) while six patients had a decrease in the burden of their associated immunosuppressants.

Additionally, Korsten et al. [93] evaluated the radiological progression and outcome measures of ILD under immunosuppressive therapy in twelve patients with ASyS, seven of whom received RTX. Most patients demonstrated stabilization of lung function and radiologic improvement on HRCT. No significant differences between the RTX and non-RTX-treated groups were detected. However, it should be noted that patients receiving RTX had significantly impaired initial pulmonary function tests (PFTs) and more frequent systemic manifestations, such as myositis and arthritis. 

Another observational study [94], compared the effectiveness of RTX versus CYC in patients with ASyS-ILD, with 34 patients receiving CYC and 28 patients receiving RTX. In the CYC and RTX groups, 88% and 54% of patients received concomitantly another immunosuppressant therapy, respectively. Pulmonary progression-free survival (PFS) was defined based on clinical features (i.e., dyspnea), pulmonary function tests (FVC and DLCO), and imaging. The CYC group exhibited worse FVC and DLco than the RTX group at inclusion, although the latter group appeared to have more refractory disease, based on the number of previous lines of immunosuppressive treatment. Although RTX and CYC demonstrated similar PFS at 6 months, RTX was associated with a better 2-year PFS, supporting its use as maintenance therapy. Finally, meta-analyses further support the role of RTX as an effective and relatively safe treatment choice in patients with IIMs, especially for refractory cases. Overall benefit in the case of lung involvement in IIM was observed in 65% of patients [95]. Regarding anti-MDA5 dermatomyositis (DM) with ILD, a meta-analysis studied the response of 35 patients to RTX, of whom 71% responded to treatment [96].

The 2023 ACR/CHEST guidelines include RTX as a first-line treatment option for patients with IIM-ILD [98]. In the recent ERS/EULAR guidelines, RTX is strongly recommended in any IIM-ILD, including patients with high risk of progression and/or severe multiorgan involvement and rapidly progressive ILD over weeks to a few months [99].

## 5. Rituximab Safety

### 5.1. Hypogammaglobulinemia

Mean immunoglobulin levels tended to decrease in patients who received RTX, particularly with repeated courses or long-term use. Short-term studies showed modest clinical impact, as mean immunoglobulin levels decreased in patients who received a single course of RTX, but only a small proportion dropped to abnormal levels [44,109]. In an open-label, double-blind, controlled study of 161 patients, Edwards et al. [45] showed that, throughout a 24-week study period, RTX treatment was associated with a near-complete depletion of peripheral-blood B cells. However, levels of immunoglobulins did not change substantially (mean values remained within normal ranges for IgG, IgM, and IgA isotypes).

Contrary to the short-term, long-term safety analyses support that repeated RTX exposure increases the likelihood of sustained hypogammaglobulinemia [110,111]. In a pooled analysis of 2578 patients who received RTX together with methotrexate in clinical trials for RA (some of whom received RTX for up to five cycles over six years), 23%, 5% and 1% of patients developed levels of IgM, IgG, and IgA below the lower normal limit (LNL), respectively. Following Course 1, 10% of patients had IgM < LNL vs. 40% following Course 5. Conversely, the proportion of patients with IgG < LNL by course remained relatively stable, with 3–6% < LNL. Serious infections occurred in 6 out of 32 patients with sustained low IgG; however, given the low number of these patients, this was not statistically significant [112]. Moreover, a cohort study of 4479 patients determined that hypogammaglobulinemia was associated with a significantly higher hazard of serious infections and mortality. However, severe infection rates were not increased significantly following treatment in the rheumatic disease subgroup [113].

Finally, late-onset neutropenia is a recognized complication of RTX therapy in patients treated for lymphoid malignancy. However, it is less frequent in patients with RA and other autoimmune diseases. In a cohort of 209 patients with rheumatic diseases, 11 patients developed late-onset neutropenia, with the incidence for patients with RA being 3% [114].

### 5.2. Rituximab and COVID-19

RTX is associated with a higher COVID-19 infection risk among patients with systemic autoimmune rheumatic diseases [115], and is associated with longer hospital stay [116]. In addition, a large nationwide cohort study in the UK showed that patients with immune-mediated inflammatory disease were at a higher risk for COVID-19-related hospital admission and death. Among them, patients treated with RTX were associated with a higher risk of COVID-19-related death compared to patients prescribed TNF inhibitors, interleukin (IL)-12/IL-23 inhibitors, IL-17 inhibitors, IL-6 inhibitors, or Janus kinase inhibitors (JAKi) [117]. Similarly, data from the COVID-19 Global Rheumatology Alliance (GRA) showed that patients with RA treated with RTX or JAKi had worse COVID-19 severity than those on TNFi [118]. In further analyses of the same registry, the baseline use of biologic or targeted synthetic DMARDs showed heterogeneity in COVID-19 severity, with RTX consistently associated with the poorest outcomes compared with other biologics [119]. Also, COVID-19 may persist or relapse in patients on B-cell-depleting biologic therapies such as RTX. A single-center, retrospective cohort study examined the rate and outcome of persistent-relapsing COVID-19 (prCOVID-19) in patients with CTDs treated with RTX. Across the prCOVID-19 findings of >30 days, along with persistently positive or PCR-based conversion in upper or lower respiratory tract samples, 26 out of 225 (11.6%) CTD patients, previously diagnosed with COVID-19 during RTX treatment period, developed 27 prCOVID-19 events [120].

Moreover, RTX-treated patients developed impaired vaccine responses, reflected in increased rates of breakthrough infections after full vaccination [121]. This reduced protection is attributable to prolonged B-cell depletion and impaired antibody production. Even in the Omicron Era, patients with systemic autoimmune rheumatic diseases treated with RTX tended to present higher odds of hospitalization [122].

### 5.3. Opportunistic Infections and Viral Reactivation

Prolonged B-cell depletion and subsequent hypogammaglobulinemia may predispose patients to opportunistic infections (OI), which can be severe or fatal. A case report described a fatal *Pneumocystis jirovecii* pneumonia following RTX administration for RA [123]. A prospective observational study of 19,282 patients commencing biologic therapy for RA estimated the risk of OI [124], with 5072 of them treated with RTX. Twenty-five had an OI, with *Pneumocystis jirovecii* pneumonia being the most common (9/25), followed by herpes zoster (7/25). The incidence of tuberculosis was significantly lower among RTX users than anti-TNF users (two documented cases). Furthermore, there were also case reports where RTX was associated with cryptococcal meningitis and cytomegalovirus colitis [125,126]. Finally, there were cases suggesting an increased risk, about 1 case per 25,000 individuals, of progressive multifocal leukoencephalopathy (PML) in RA patients treated with RTX. PML presented as a progressive neurological disorder, with diagnosis confirmed by detection of JC virus DNA (Human polyomavirus 2, *commonly referred to as the JC virus*) in the cerebrospinal fluid or brain biopsy specimen [127].

RTX therapy in RA has also been associated with the reactivation of the hepatitis B virus (HBV), a potentially life-threatening complication [128]. However, this complication was much more common in patients with hematologic malignancies and significantly less frequent among patients receiving RTX for the treatment of rheumatic diseases [129]. A small cohort of 54 RA patients who received RTX found that HBV reactivation occurred not only in HBsAg-(+) patients but also in those with resolved infection (HBsAg(-)/HBcAb(+)) [130]. Consequently, all patients should be screened for HBsAg and anti-HBc prior to starting treatment, and prophylactic treatment is recommended for patients who are positive for HBsAg and/or anti-HBc [131]. Although uncommon, rituximab may also predispose to de novo HBV infection. B-cell depletion can lead to a decline or loss of protective anti-HBs titers in previously vaccinated individuals, potentially rendering them susceptible to primary infection. A representative case was described by Westhoff et al., in which a patient with initially protective anti-HBs developed acute HBV infection after RTX [132].

## 6. Conclusions

Lung involvement in the CTD setting poses a significant burden on quality of life, morbidity, and mortality. Thus, prompt diagnosis and optimal management are crucial. B cells play a multifaceted role in the pathogenesis of CTD-ILD through a plethora of different mechanisms, including both antibody-dependent and antibody-independent pathways. The common finding of ectopic lymphoid aggregates and GC in the lungs in patients with CTD-ILD highlights their central role in ongoing inflammation and subsequent fibrosis. RTX, through its B-cell-depleting activity, has demonstrated promising efficacy across the spectrum of CTD-ILD. Its therapeutic efficacy is underlined in the recent ACR/CHEST and ERS/EULAR guidelines. Further studies are needed to better define predictive markers, refine patient selection, and determine the optimal timing and duration of therapy to maximize clinical benefit and minimize adverse events.

## Figures and Tables

**Table 1:** Mechanisms of action and modes of regulation of Rituximab.

Mechanism of Action	Modes of Regulation
Antibody-Dependent Cellular Cytotoxicity (ADCC)	Recruitment of effector cells; cytotoxic events through binding to Fcγ receptor NK-cell engagement; perforin-mediated apoptosis Macrophages and neutrophils participate through FcγR-mediated cytotoxicity and phagocytosis Rituximab-opsonized B cells act as decoy immune complexes that activate phagocytic capacity of monocytes
Complement-Dependent Cytotoxicity	Binding to C1q; activation of classical complement cascade Fragments of C3a and C5a function as anaphylatoxins to further recruit effector cells Binding of C5a to C5aR on local macrophages renders them activated Amplification to ADCC through CR3 binding to iC3b ligands
Induction of Apoptosis-Caspase-dependent	Activation of Src family tyrosine kinases Activation of Fas-apoptotic signaling Inhibition of major survival pathways (e.g., p38 MAPK, ERK1/2, NF-κB, and Akt)
Decreased autoantibody production	Autoantibody decline is observed post-treatment with rituximab Dampening of disease-specific autoantibodies linearizes with positive clinical response (RF/anti-CCP in RA, ANCAs in AAVs, anti-dsDNA in SLE)
Reduced Antigen Presentation	Disruption of antigen presentation mechanisms by B-cell depletion Downregulation of CD40/CD80 on B-cells abrogates co-stimulatory signaling on T cells Due to B-cell depletion and loss of co-stimulation, T cells express lower levels of CD69, HLA-DR, and IL-17, and regulatory T cells are restored
Modulation of Cytokine Milieu	Reduction in pro-inflammatory cytokines such as IL-6, IL-7, and TNF-α Boost of anti-inflammatory cytokines, such as IL-10 Dampening of T-cell-driven inflammation through decreasing IFN-gamma and IL-1β levels Reprogramming of matrix metalloproteinase-mediated inflammation
Modulation of Ectopic Lymphoid Structures	Regression of lymphoid infiltrates and granulomatous formations Remodeling of ELS microarchitecture; disruption of cellular composition/interactions (B-/T-cell subtypes and balance) Reduction in disease-specific autoantigens due to ELS collapse
Fibroblast Phenotype	Normalization of fibroblast phenotype and reduction in type I collagen production Blockade of fibroblast stimulation by a decrease in anti-PDGFR autoantibodies Decreased dermal thickness and myofibroblast density in skin biopsies of SSc patients post-treatment with rituximab; improvement in lung function Inhibition of Wnt pathway through upregulation of Dkk-1 expression suppresses fibrotic procedure

Abbreviations: NK—natural killer, ADCC—antibody-dependent cellular cytotoxicity, C—complement, ELS—ectopic lymphoid structure, RA—rheumatoid arthritis, SSc—systemic sclerosis, PDGFR—platelet-derived growth factor receptor.

**Table 2:** Summary of rituximab studies in CTD-ILDs.

Disease	Study (First Author, Year)	Study Design	Number of Patients	Main Findings	Comments
RA-ILD	Md Yusof et al., 2017 [77]	Observational retrospective cohort study	44	23 (56%) were stable, and 7 (16%) had improved, while 14 (32%) showed progression, with 9 deaths (16%)	Progression was defined by decrease in FVC > 10% or DLCO > 15%, or radiological deterioration in HRCT, or death from progressive ILD
RA-ILD	Vadillo et al., 2020 [78]	Longitudinal multicenter observational study	68	Patients exposed to RTX had a higher probability of remaining free of PFT impairments compared to other therapies.	Functional impairment was defined as a relative decline of FVC > 5%.
RA-ILD	Bopana et al., 2024 [79]	Systematic review and meta-analysis	314	Patients under RTX treatment exhibited an improvement in FVC from baseline by 7.5%	RTX improved DLCO by 6.39%
RA-ILD	Kelly et al., 2021 [80]	Retrospective multicenter observational study	290	Patients receiving RTX as their first biologic factor had longer three- (92%), five- (82%), and seven-year (80%) survival than those whose first biologic treatment was an anti-TNF agent.	Relative risk (RR) of death from any cause was increased among patients who had received anti-TNF therapy, while RR was lower in those treated with RTX or MMF.
RA-ILD	Druce et al., 2017 [81]	Prospective observational comparative study	43 patients on RTX vs. 309 on TNFi	Patients who received RTX had lower mortality rates than those who received TNF inhibitors.	-
RA-ILD	Saleem et al., 2024 [82]	Randomized open-label study	95	Both RTX and CYC groups demonstrated an improvement in FVC from baseline.	No statistically significant difference between the two groups at 24 and 48 weeks of FVC measurement
RA-ILD	Narváez et al., 2020 [83]	Retrospective observational study	31	RTX appears to be an effective rescue therapy	
SSc-ILD	Goswami et al., 2020 [84]	Meta-analysis (20 studies)	575	FVC improved by 4.49% at 6 months and 7.03% at 12 months; DLCO improved by 3.47% and 4.08% at 6 and 12 months, respectively.	Fewer infectious adverse events vs. other immunosuppressants.
SSc-ILD	Macrea et al., 2024 [85]	Meta-analysis (3 studies)	Not specified	Supported stabilization or improvement of pulmonary function with RTX.	
SSc-ILD	Maher et al., 2023 [73]	Double-blind, double-dummy RCT (RTX vs. CYC)	110 (mixed CTD-ILD: 38% SSc, 45% IIM, 16% MCTD)	Similar efficacy to CYC; improvements in FVC at 24 and 48 weeks	Consistent across CTD-ILD subgroups.
SSc-ILD	Sircar et al., 2018 [86]	Open-label RCT (RTX vs. CYC)	60	FVC (mean %) improved from 61.30 to 67.52 with RTX; declined from 59.25 to 58.06 with CYC.	More serious adverse events occurred in the CYC group.
SSc-ILD	Yan et al., 2025 [87]	Post hoc analysis	955	No statistical difference in change in FVC between RTX, MMF, and tocilizumab.	Large registry-based comparison.
SSc-ILD	Daoussis et al., 2017 [88]	Multicenter open-label comparative study	51	RTX group: increase in FVC at 2 years Control: no change.	Compared RTX to conventional treatment.
SSc-ILD	Daoussis et al., 2010 [89]	Prospective comparative	14 (8 RTX; 6 control)	At 1 year, RTX group had better FVC and DLCO; at 2 years, FVC increased from 68.13 to 77.13 (mean ± SEM).	Limited sample; long-term follow-up.
IIM-ILD	Bauhammer et al., 2016 [90]	Retrospective observational study	11	Improvement in 9/11 patients, complete response in 1 patient, and 1 death due to pneumonia. Response is also seen in anti-Ro52-positive cases.	High efficacy in refractory anti-Jo-1 ASyS-ILD.
IIM-ILD	Conticini et al., 2024 [91]	Prospective observational study	18	No deterioration in lung function during follow-up after prednisone tapering.	RTX is effective as maintenance after MMF or MTX failure.
IIM-ILD	Allenbach et al., 2015 [92]	Open-label Phase II trial	10	Improvement in 5/10, stabilization in 4/10, worsening condition in 1/10. Glucocorticoid dose reduced from 52.5 mg/day to 9 mg/day.	Refractory ASyS-ILD; decreased need for other immunosuppressants.
IIM-ILD	Narvaez et al., 2024 [83]	Observational retrospective study	28	FVC and DLCO decline reversed at 6 months, sustained benefit at 1 and 2 years.	Decreased steroid dose and oxygen dependence.
IIM-ILD	Korsten et al., 2021 [93]	Observational, comparative study	12 (7 RTX)	Most RTX-treated patients had stable PFTs and improved HRCT.	RTX group had more severe baseline disease.
IIM-ILD	Langlois et al., 2020 [94]	Retrospective comparative study, RTX vs. CYC	28 RTX vs. 34 CYC	Similar PFS at 6 months; RTX had better 2-year pulmonary PFS.	RTX is associated with better long-term outcomes.
IIM-ILD	Zhen et al., 2022 [95]	Meta-analysis (26 studies)	—	Pooled efficacy for IIM-ILD ≈ 65% improvement or stabilization.	RTX is generally safe, especially for refractory disease.
IIM-ILD	He et al., 2021 [96]	Meta-analysis (anti-MDA5 DM)	34	71% response rate; skin rash improved in >50%. Infections in 37% of cases.	Supports benefit in anti-MDA5-positive DM-ILD.
CTD-ILD	RECITAL, Maher et al., 2023 [73]	Double-blind, double-dummy, randomized, controlled, phase 2b trial	145	RTX was not superior to CYC in treating patients with CTD-ILD, although participants in both treatment groups had increased FVC at 24 weeks	Participants between 18 and 80 years with severe or progressive ILD related to SSc, IIM, or MCTD were randomly assigned (1:1) to receive RTX or CYC
CTD-ILD	EVER-ILD, Manikian et al., 2022 [74]	Randomized, double-blind, two-parallel group, placebo-controlled trial	112	Combination of RTX and MMF was superior to MMF alone in patients with ILD and an NSIP pattern	Patients with connective tissue disease-associated ILD or idiopathic interstitial pneumonia (with or without autoimmune features) and an NSIP pattern, in a 1:1 ratio, to receive RTX (1000 mg) or placebo on day 1 and day 15 in addition to MMF (2 g daily) for 6 months.
CTD-ILD	Sharp et al., 2016 [76]	Retrospective observational study	24	RTX improves FVC and stabilizes DLCO.	-

Abbreviations: CTD—connective tissue disease, MCTD—mixed connective tissue disease, ILD—interstitial lung disease, FVC—forced vital capacity, DLCO—diffusing capacity for carbon monoxide, PFTs—pulmonary function tests, RTX—rituximab, CYC—cyclophosphamide, RA—rheumatoid arthritis, SSc—systemic sclerosis, IIM—idiopathic inflammatory myopathy, PFS—progression-free survival, ASyS—antisynthetase syndrome, TNFi—tumor necrosis factor inhibitor, MMF—mycophenolate mofetil, MTX—methotrexate, NSIP—non-specific interstitial pneumonia.

## Data Availability

No new data were created or analyzed in this study. Data sharing is not applicable to this article.
